# Supplementary material for: Parallel, Continuous Monitoring and Quantification of Programmed Cell Death in Plant Tissue
Source: Adv Sci (Weinh). 2024 Mar 26;11(23):2400225. doi: 10.1002/advs.202400225 (PMC11187890; doi:10.1002/advs.202400225)
Supplement: Supplementary file 1 — Supporting Information [file ADVS-11-2400225-s001.pdf]

ADVANCED SCIENCE
Open Access

Supporting Information

for *Adv. Sci.*, DOI 10.1002/advs.202400225

Parallel, Continuous Monitoring and Quantification of Programmed Cell Death in Plant Tissue

*Alexander Silva Pinto Collins, Hasan Kurt, Cian Duggan, Yasin Cotur, Philip Coatsworth, Atharv Naik, Matti Kaisti, Tolga Bozkurt and Firat Güder**

Supporting Information

Parallel, Continuous Monitoring and Quantification of Programmed Cell Death in Plant Tissue

*Alexander Silva Pinto Collins, Hasan Kurt, Cian Duggan, Yasin Cotur, Philip Coatsworth, Atharv Naik, Matti Kaisti, Tolga Bozkurt, Firat Güder**

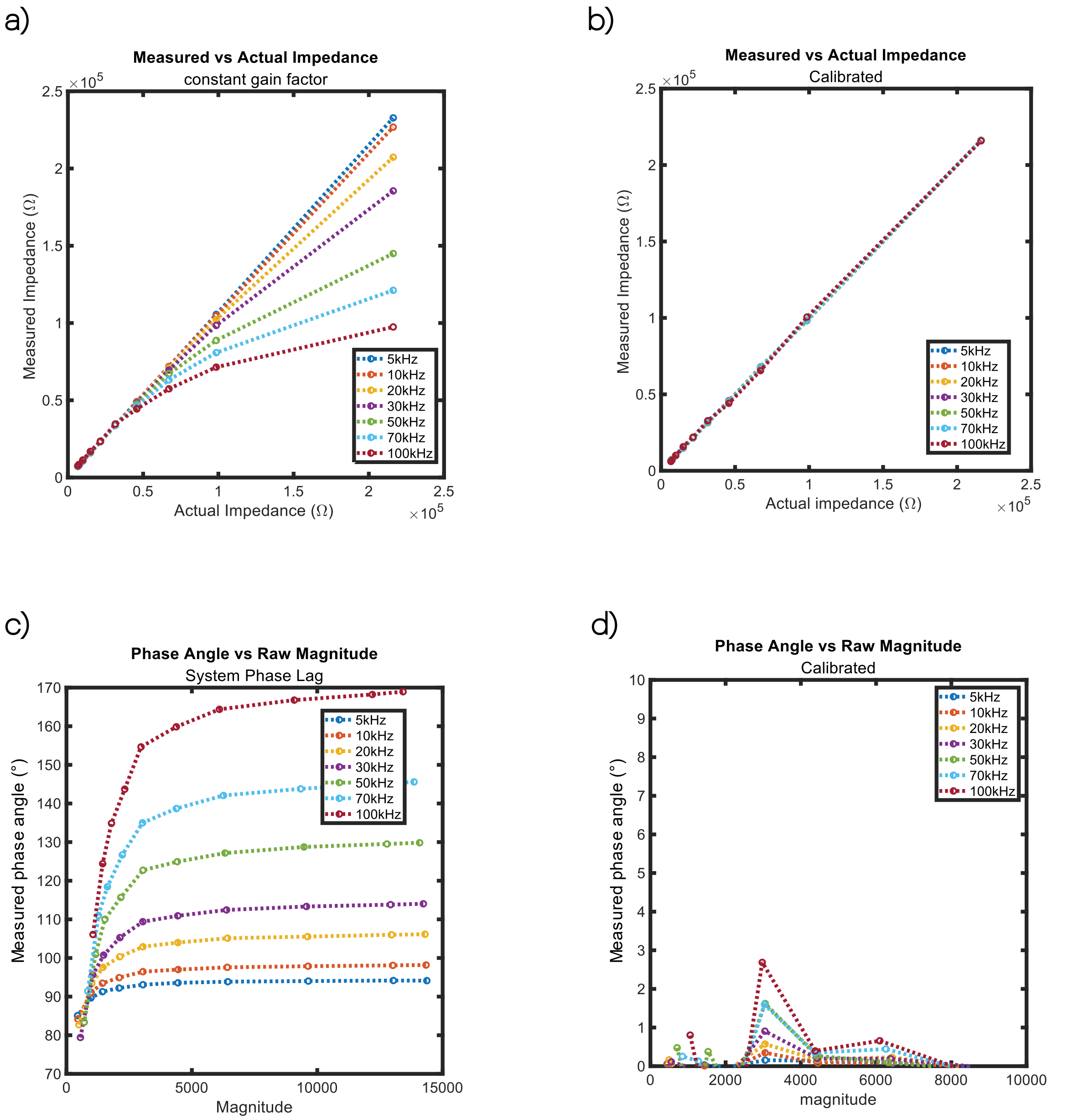

Figure S1. Gain factor and Phase Angle System Calibration: a) Correlation plot of measured and actual impedance, measuring resistances in the range 6.6-220 kΩ using the PASTEL system. Impedance calculated using constant gain factor calculated as described in **Equation S2 and Equation S3. b)** Correlation plot of measured and actual impedance, measuring resistances in the range 6.6-220 kΩ using the PASTEL system. Impedance calculated using 4th order polynomial mapping function (**Equation S4**), derived from application of a fitting function performed on data obtained in a). **c)** Correlation plot of system phase angle against raw magnitude measured, measuring resistances in the range 6.6-220 kΩ using the PASTEL system. **d)** Correlation plot of measured phase angle against raw magnitude measured, measuring resistances in the range 6.6-220 kΩ using the PASTEL system. Measured Phase angle calculated as described in **Equation S5** using a power mapping function derived from application of a fitting function performed on data obtained in c).

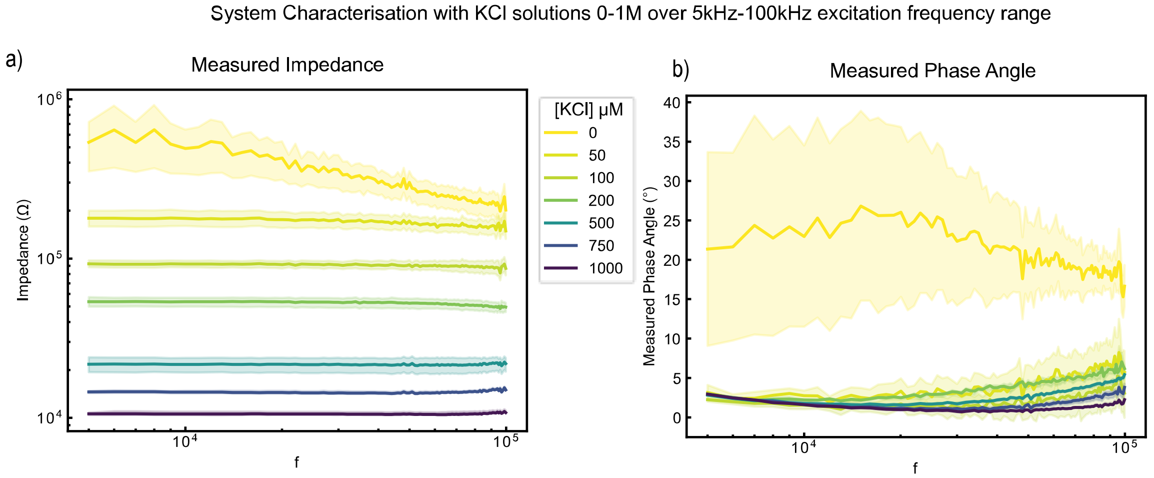

Figure S2. System Characterization with Potassium Chloride (KCl): **a)** Impedance measured with PASTEL using range of KCl concentrations (0 — 1.0 M) against excitation frequency Data captured at 20 minutes. Data represented as μ ± σ, with n = 3 independent samples **b)** Phase angle measured with PASTEL using range of KCl concentrations (0 — 1.0 M) against excitation frequency Data captured at 20 minutes. Data represented as μ ± σ, with n = 3 independent samples.

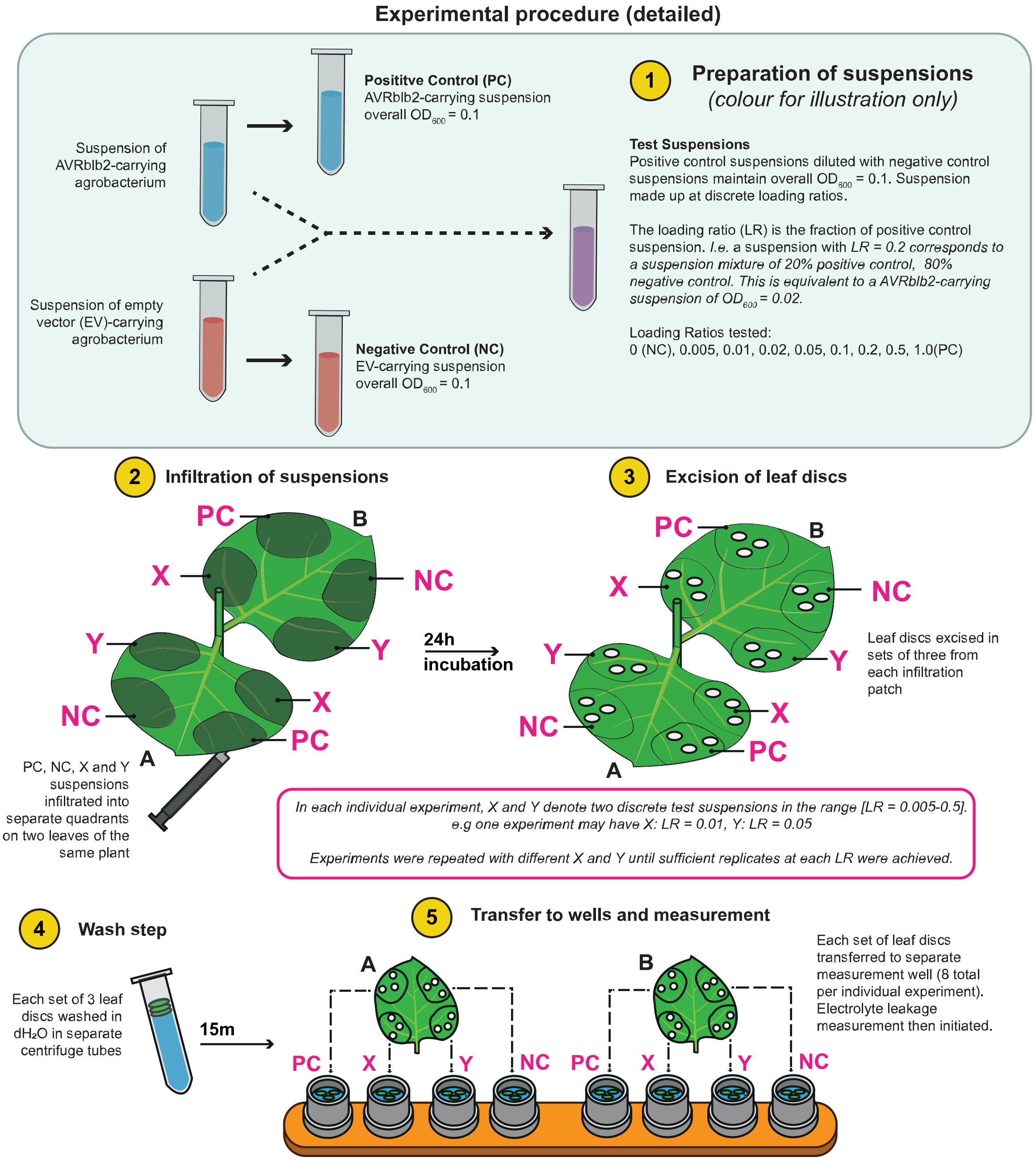

Figure S3: Detailed experimental procedure. 1) Preparation of test suspensions for eliciting differing intensities of HR. All suspension have an OD_{600} = 0.1 to control for any plant response caused by the bacteria itself. 2) Infiltration diagram for a single experiment, using two leaves from a single plant. 3) Excision of leaf discs. 4) Wash step to remove compounds released by excision. 5) Transfer of leaf discs to measurement setup. Each well contains the leaf discs from one infiltration patch.

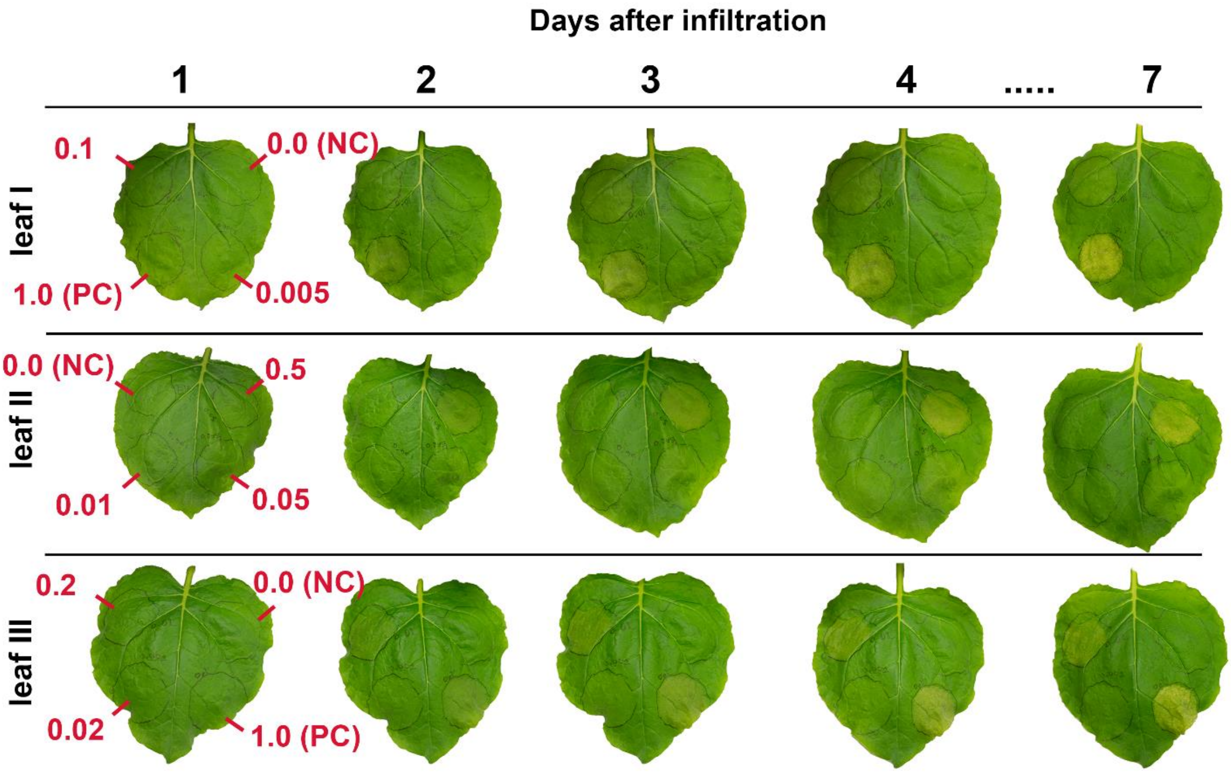

Figure S4. Visual HR Symptoms at 1-4 and 7 days after infiltration. Images of leaves agroinfiltrated with an empty vector-carrying bacterial suspension (negative control) and AVRblb2-carrying bacterial suspensions of loading ratios in the range LR= 0.005 - 1.0.

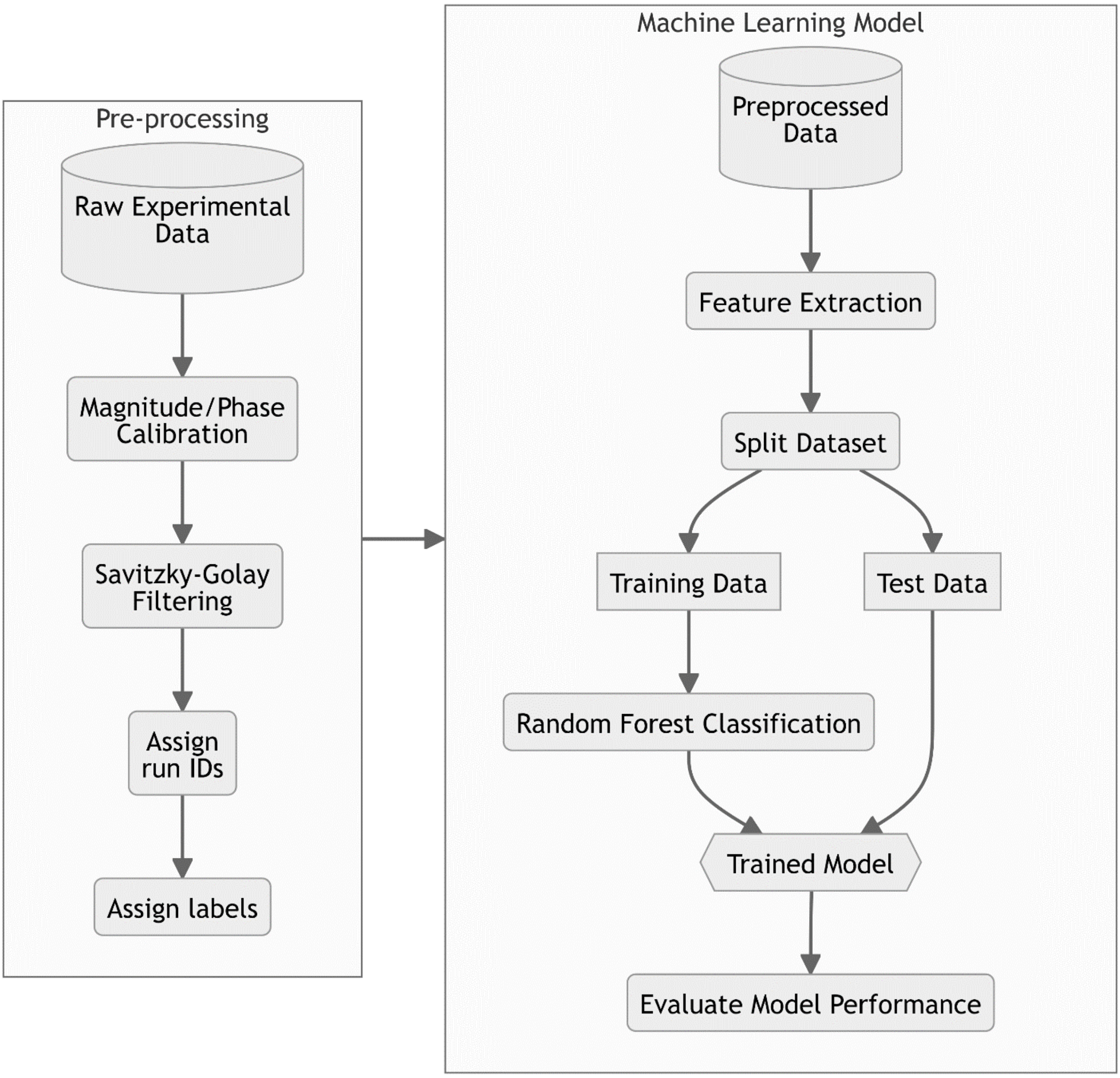

Figure S5. Flowchart outlining data pre-preprocessing steps and development of machine learning models for classification of HR

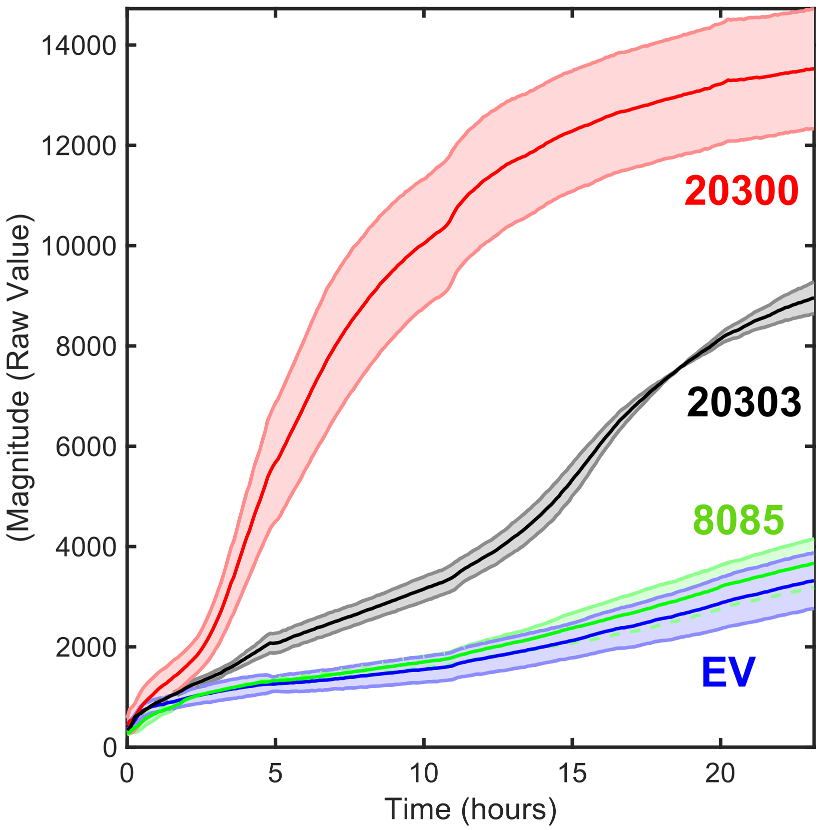

Figure S6. Electrolyte leakage experiment with AVRblb2 paralogs conducted with an earlier version of PASTEL. A leaf of Rpi-blb2 transgenic *N.benthamiana* was infiltrated with suspensions carrying constructs of EV and AVRblb2 paralogs PITG_20300, PITG_20303 and PITG_8085 at OD_{600} = 0.1. PITG_20300 is the reference paralog used in all other experiments. One leaf disc was placed in each well (an Eppendorf tube) in 1ml dH_2O. Data n=2, shaded region represents max and minimum values.

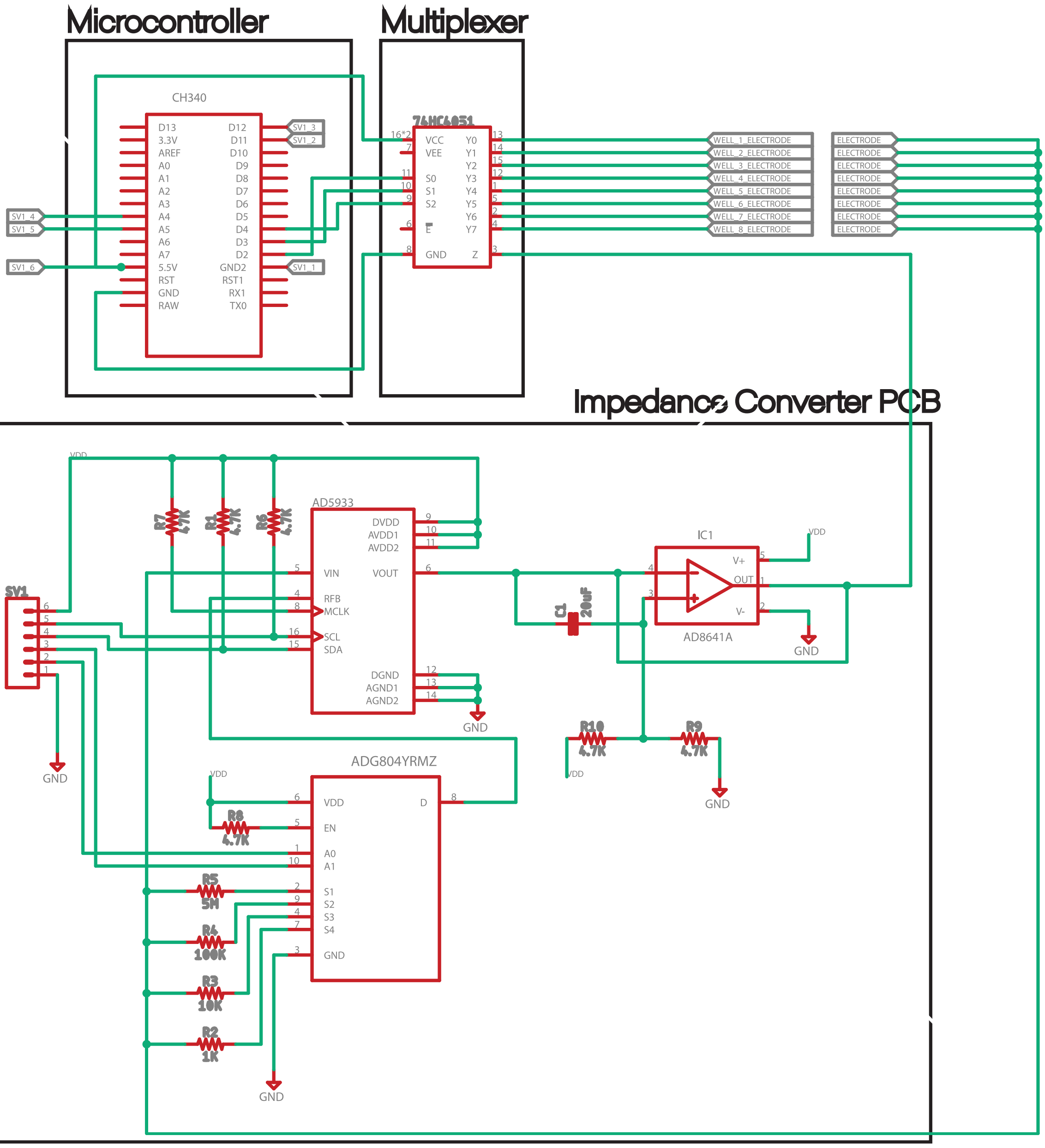

Figure S7. System electronics schematic of microcontroller, multiplexer and custom printed circuit board.

Equation S1: Conversion of Raw Magnitude (at 10kHz) to Conductivity

$$\sigma = \gamma\, M_{\mathrm{PASTEL}}$$

$$\gamma = \bar{\sigma}_{\mathrm{HANNA}(500\ \mu\mathrm{M\ KCl})} / \bar{M}_{PASTEL(500\mu\mathrm{M}\ KCl)}$$

$$\gamma = 85.7\ /\ 4869.32$$
$$\gamma = 0.0176\ \mu S \cdot cm^{-1}$$

where γ = scale factor, σ = conductivity and M_{PASTEL} = raw magnitude measured by PASTEL.

Constant scale factor can be used at for measurements performed with 10 kHz excitation frequency as system measurements are linear throughout the relevant impedance range at this frequency (**Figure S2a**).

Equation S2: Recommended Calibration Resistance[1]

$$R_{CAL} = \frac{Z_{min} + Z_{max}}{3}$$

$$75.5k = (220k + 6.6k)/3$$
$$\rightarrow\ 67\ k\Omega$$

Equation S3: Constant Gain Factor Calculation[2]

$$Gain\ Factor = Magnitude_{50kHz}/R_{CAL}$$
$$\mathrm{Gain\ Factor} = 1164665.86/67000$$
$$Gain\ Factor = 9.70579e - 9\ \Omega^{-1}$$
$$Magnitude = \sqrt{(R^2 + I^2)}$$

where R = Real and I = Imaginary data word outputs from the AD5933 Impedance Converter.

Equation S4: Impedance calibration[2]

$$Z(x, f) = a_f x^3 + b_f x^2 + c_f x + d_f$$

where Z is the calibrated impedance, f is frequency, $x = \frac{Gain\ Factor}{Raw\ Magnitude}$, and a_f, b_f, c_f, d_f are frequency dependent constants.

Equation S5: Phase Angle calibration[2]

$$Z\emptyset\ =\ \Phi\ -\ \nabla$$

where $Z\emptyset$ is the phase of the unknown impedance, Φ is the raw phase angle and ∇ is the system phase angle

$$\nabla(M, f) = a_f e^{b_f M} + c_f e^{d_f M}$$

where ∇ is the system phase angle, M is the raw magnitude, and a_f, b_f, c_f, d_f are frequency dependent constants.

Table S1. Statistical analysis of fluorescence quantification from Propidium Iodide fluorescence imaging. Each datapoint corresponds to an individual z-stack of images on the x-y plane. Quantification performed by projecting maximum intensity across the Z-stack and calculating the percentage of pixels above a fixed threshold intensity. a) One-way ANOVA for 0 hour (24h post infiltration) groups b) Post-hoc Dunnett's test for 0 hour (24h post infiltration) groups c) One-way ANOVA for 24 hour (48h post infiltration) groups d) Post-hoc Dunnett's test for 24 hour (48h post infiltration) groups.

**(a) One-way ANOVA : 0 hours (24h post infiltration)**					
**Source of variation**	**Sum of squares**	**Degrees of freedom**	**Mean squared error**	**F-statistic**	**p-value**
Between Groups	43.7754	2	21.8877	60.3	3.08E-11
Error	10.8885	30	0.363		
Total	54.6639	32			
**(b) Post-hoc Dunnett's test : 0 hours (24h post infiltration)**					
**Group**	**Control Group**	**Mean Difference**	**p-value**	**Lower Bound**	**Upper Bound**
0.005	0.0 (NC)	0.016174962	0.996943806	-0.570796537	0.603146462
1.0 (PC)	0.0 (NC)	2.515254902	4.40E-10	1.891175613	3.139334192

**(c) One-way ANOVA : 24 hours (48h post infiltration)**					
**Source of variation**	**Sum of squares**	**Degrees of freedom**	**Mean squared error**	**F-statistic**	**p-value**
Between Groups	296.601	2	148.301	122.56	3.67E-15
Error	36.301	30	1.21		
Total	332.903	32			
**(b) Post-hoc Dunnett's test : 24 hours (48h post infiltration)**					
**Group**	**Control Group**	**Mean Difference**	**p-value**	**Lower Bound**	**Upper Bound**
0.005	0.0 (NC)	0.46134284	0.51128559	-0.606257153	1.528942834
1.0 (PC)	0.0 (NC)	6.730679738	1.25E-14	5.635583399	7.825776078

Table S2. Table showing the breakdown of system cost and consumables per experiment sample. *All prices are in USD.*

**System**		
**Component**	**Supplier**	**Price (USD)**
ATmega328P CH340 Nano	Kunkune	6.27
AD5933YRSZ	Digikey	22.69
AD8641A	Digikey	4.98
ADG804YRMZ	Digikey	4.66
Sparkfun 74HC4051	Pimoroni	3.46
PCB Manufacturing + SMD Components	Elecrow	6.4
ABS Filament (8 Wells)	Verbatim	~3.84
PLA Filament (Well Holder)	RS	~2.56
**Total**		**~54.86**
**Consumables (per well)**		
SparkFun Electronics Jumper PRT-12794	Digikey	0.21
Greiner Bio-one 1.5ml Reaction Tube cap, 616-201	Greiner Bio-one	~0.03
TE Connectivity PCB Mount Header 5-826634-0	Mouser	0.23
RS PRO Nitrile Rubber O-Ring, 6.5mm Bore, 10.5mm Outer Diameter	RS	0.06
**Total**		**~0.53**

Supporting Video
Video S1. Animated representation of the PASTEL sample preparation and HR measurement procedures.

Supplementary References
[1] Analog Devices Inc., AN-1252 Technical Note, http://www.analog.com/media/en/technical-documentation/application-notes/AN-1252.pdf
[2] Analog Devices Inc., AD5933 Datasheet, https://www.analog.com/media/en/technical-documentation/data-sheets/AD5933.pdf
